# Case Report: The silent giant: biochemical-clinical dissociation in a large cystic pheochromocytoma

**DOI:** 10.3389/fonc.2026.1770384

**Published:** 2026-03-11

**Authors:** Jing Zhang, Qingli Zhao, Jin Wang, Xiaoqing Yang, Shengliang Huang

**Affiliations:** 1 Department of Pathology, The First Affiliated Hospital of Shandong First Medical University and Shandong Provincial Qianfoshan Hospital, Jinan, Shandong, China; 2 Department of Urology, The First Affiliated Hospital of Shandong First Medical University and Shandong Provincial Qianfoshan Hospital, Jinan, Shandong, China

**Keywords:** adrenal incidentaloma, asymptomatic pheochromocytoma, biochemical-clinical dissociation, preoperative alpha-blockade, SDHB deficiency

## Abstract

**Background:**

Asymptomatic pheochromocytomas are increasingly detected as incidentalomas. However, large tumors presenting with marked biochemical elevation but complete clinical silence (“biochemical-clinical dissociation”) are rare and prone to catastrophic mismanagement if mistaken for non-functional masses.

**Case presentation:**

We report a case of a 43-year-old normotensive female presenting with a large (57×53 mm) incidental left adrenal mass. Abdominal imaging revealed a cystic-solid tumor with intratumoral hemorrhage. Despite the complete absence of sympathetic symptoms, biochemical evaluation showed markedly elevated plasma normetanephrine levels (1168.0 pg/ml), indicating significant secretory activity. The patient was successfully managed with preoperative alpha-blockade (doxazosin) and volume expansion, followed by an uneventful transperitoneal laparoscopic adrenalectomy. Histopathology confirmed a pheochromocytoma with loss of SDHB expression.

**Conclusion:**

This suggests a multifactorial mechanism involving cystic sequestration and biochemical alterations associated with SDHB deficiency. Clinicians must recognize that biochemistry, not symptoms, dictates management. Mandatory preoperative α-blockade remains the cornerstone for preventing lethal intraoperative hemodynamic crises in these “silent” yet biochemically active tumors.

## Introduction

Pheochromocytomas are increasingly detected as asymptomatic incidentalomas due to widespread imaging (1, 2). However, a distinct phenotype presenting with “biochemical-clinical dissociation”—marked catecholamine elevation despite complete clinical silence—remains a perilous diagnostic pitfall (3). This presentation is exceedingly rare; while adrenal incidentalomas are common, those exhibiting biochemical activity without symptoms represent a small fraction of cases, often leading to delayed or incorrect management. Misinterpreting this silence as tumor inactivity can lead to fatal intraoperative hemodynamic crises. Herein, we report a rare case of a giant cystic pheochromocytoma exhibiting this paradox. We identify the loss of SDHB expression as the pathogenic driver, elucidating a novel mechanism where pseudohypoxia-induced angiogenesis leads to “cystic sequestration” of hormones. This case underscores that biochemistry, not symptoms, must dictate preoperative management to prevent lethal complications.

## Case presentation

A 43-year-old female was referred to The First Affiliated Hospital of Shandong First Medical University and Shandong Provincial Qianfoshan Hospital (Jinan, China) in February 2023 for an incidentally discovered left adrenal tumor. The patient was entirely asymptomatic, with no history of hypertension, palpitations, or sweating. She had no significant past medical or family history and was normotensive upon examination.

**Figure 1:**
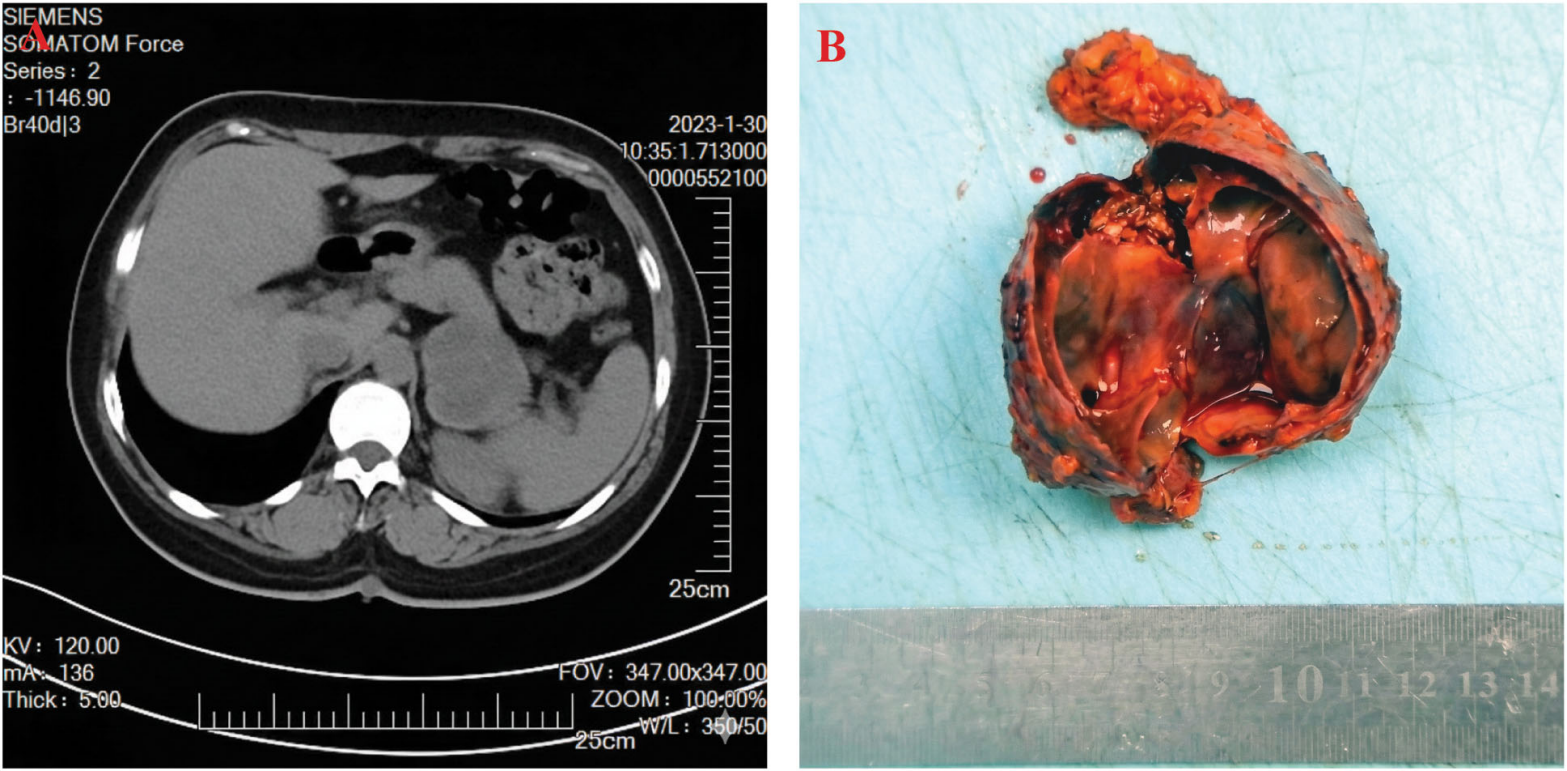
**(A)** Contrast−enhanced CT demonstrates a large cystic−solid left adrenal mass measuring 57 × 53 mm, with internal septa, showing slight and heterogeneous enhancement. **(B)** The resected specimen is well circumscribed, with a cystic cut surface, gray−yellow in color, and focal hemorrhage.

Computed tomography (CT) revealed a large, 57×53 mm, cystic-solid left adrenal mass with internal septa on non-contrast imaging. After contrast administration, the tumor demonstrated slight, heterogeneous enhancement (**Figure 1A**). Although these imaging findings were nonspecific, the presence of cystic-solid components raised suspicion for pheochromocytoma with intratumoral hemorrhage or cystic degeneration.

Consequently, biochemical evaluations were performed. The patient’s plasma normetanephrine level was markedly elevated at 1168.0 pg/ml (reference range, <145 pg/ml), with a slightly elevated metanephrine level of 84.6 pg/ml (reference range, <62 pg/ml). These findings were diagnostic for pheochromocytoma. Her plasma catecholamine levels and 24-hour urinary vanillylmandelic acid (VMA) levels were within normal limits. Re-evaluation of the patient’s medication history ruled out the use of tricyclic antidepressants, alpha-blockers, and labetalol, agents typically associated with biochemical false positives. Although plasma 3-methoxytyramine was omitted from the initial workup, it was accounted for during subsequent clinical analysis.

Despite her normotensive state, preoperative management was initiated with the α-blocker doxazosin and intravenous hydration for one week to prevent perioperative hemodynamic instability. A systemic workup, including chest CT and physical examination, was performed to rule out extra-adrenal paragangliomas or other SDHB-associated malignancies, which was negative. The patient underwent a transperitoneal laparoscopic left adrenalectomy. A critical surgical step involved early ligation of the adrenal vein prior to tumor manipulation to minimize catecholamine release and hemodynamic fluctuations. The patient remained hemodynamically stable throughout the procedure, with no hypertensive episodes or subsequent hypotension. The tumor was completely resected, measuring 60x50x30 mm.

Gross examination of the resected specimen revealed significant hemorrhage and degenerative cystic changes on the cross-section, consistent with the preoperative CT findings (**Figure 1B**). Histopathology demonstrated.

**Figure 2:**
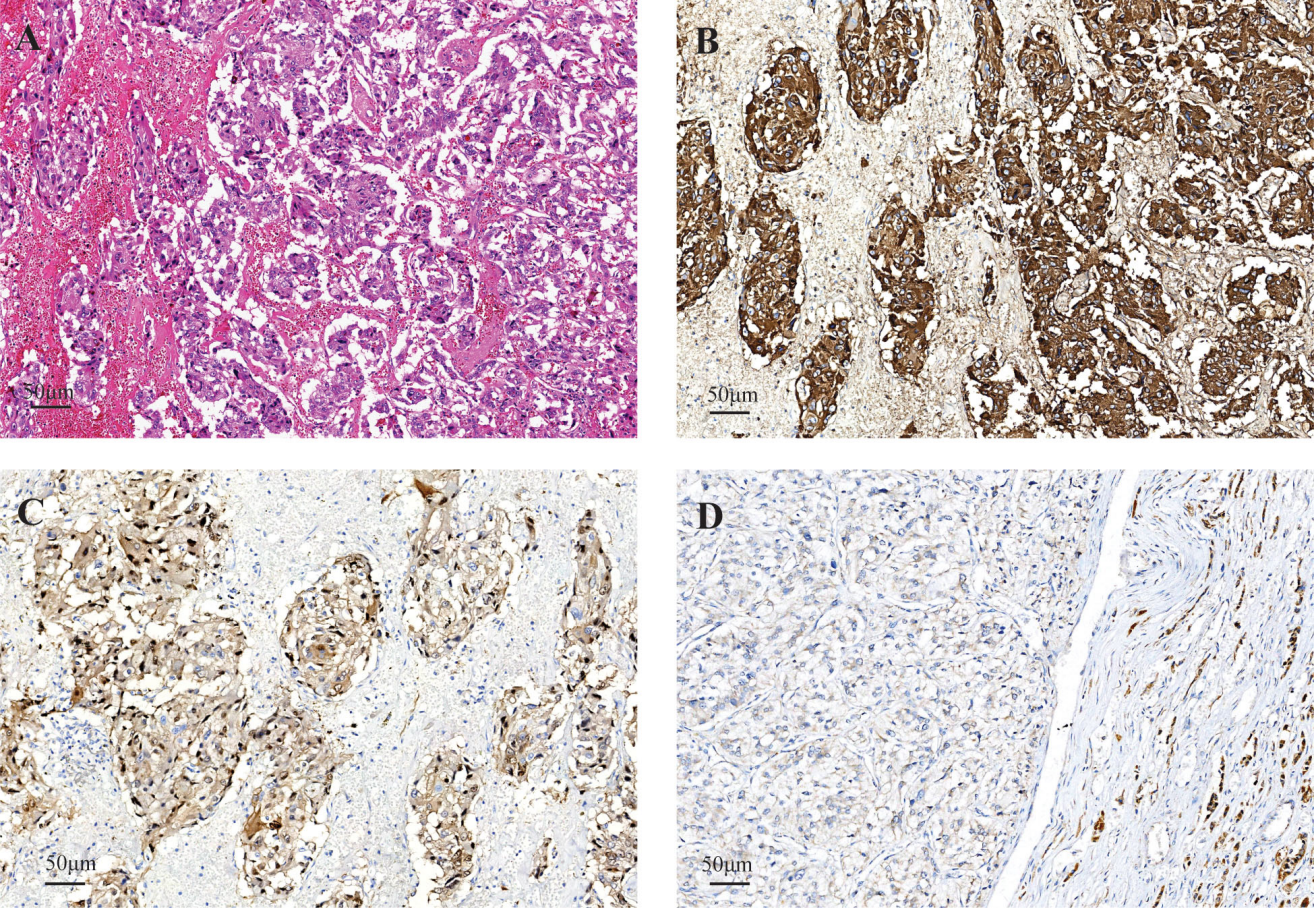
Histopathological findings of the tumor. **(A)** Tumor cells are arranged in Zellballen with mild nuclear atypia; interstitial hemorrhage is present in the left portion of the image (H&E, ×200). **(B)** Immunohistochemistry shows chromogranin A positivity in chromaffin cells and S−100 protein positivity in sustentacular cells **(C)** SDHB is negative **(D)**.

Microscopically, the tumor exhibits an overall zellballen or trabecular arrangement, with focal areas of hemorrhage and cystic change (**Figure 2A**). Cytologically, the polygonal cells contained basophilic and vesicular cytoplasm. Immunohistochemical (IHC) staining showed the chromaffin cells were positive for neuroendocrine marker chromogranin A (**Figure 2B**), S-100 protein staining highlighted the surrounding sustentacular cells (**Figure 2C**). These histopathologic findings confirmed the diagnosis of pheochromocytoma. In addition, SDHB immunohistochemistry was performed, and the result was negative (**Figure 2D**). Based on the Pheochromocytoma of the Adrenal Gland Scaled Score, the tumor received a total score of 6 points for its histopathologic features.

The patient had an uneventful recovery and was discharged 8 days postoperatively. We implemented an active follow-up strategy with biochemical testing and CT scans performed twice in the first year and once per year in the second year; to date, no evidence of recurrence has been found.

## Discussion

Asymptomatic pheochromocytoma (A-PHEO) is increasingly detected as an incidentaloma (4–6); however, our case illustrates a distinct and perilous subtype characterized by “biochemical-clinical dissociation.” Although the precise incidence of complete clinical silence in the case of an eight-fold increase in normetanephrine remains poorly defined, this phenotype represents a treacherous diagnostic pitfall (7). The absence of symptoms may lead clinicians to misclassify the mass as a non-functional incidentaloma, potentially resulting in catastrophic intraoperative hemodynamic instability if mandatory preoperative pharmacological preparation is omitted.The pivotal pathological finding in this case was the loss of SDHB expression on immunohistochemistry. This result serves as a definitive surrogate marker for SDHx mutations, firmly categorizing the tumor into Cluster 1 (the pseudohypoxia pathway) (8). The loss of SDHB function leads to the stabilization of hypoxia-inducible factors (HIFs), creating a state of “pseudohypoxia” (9, 10). This molecular background provides several complementary explanations for the clinical paradox.

First, dopaminergic shift and biochemical phenotype. Cluster 1 tumors typically lack Phenylethanolamine N-methyltransferase (PNMT) expression, preventing the conversion of norepinephrine to epinephrine (11, 12). This aligns precisely with our patient’s phenotype of exclusive normetanephrine elevation with normal metanephrine levels. Furthermore, SDHB-deficient tumors often exhibit a ‘dopaminergic phenotype.’ Although plasma methoxytyramine was not measured, the overproduction of dopamine-a potent vasodilator-may have antagonized the vasoconstrictive effects of norepinephrine, potentially contributing to the patient’s normotensive state.

Secondly cystic sequestration and angiogenesis. the loss of SDHB provides a mechanistic explanation for the tumor’s morphology and clinical silence. The activation of the pseudohypoxia pathway is known to upregulate angiogenic factors, particularly VEGF, leading to aberrant and fragile neovascularization (13–15). We postulate that this dysregulated angiogenesis precipitated the massive spontaneous hemorrhage and cystic degeneration observed in our case. This process likely resulted in “cystic sequestration,” where catecholamines were physically isolated within the cystic spaces, preventing their systemic release and maintaining hemodynamic stability. Thus, the clinical silence was not due to tumor inactivity, but rather a structural containment driven by the underlying SDHB-deficient pathology.

Thirdly adrenergic receptor desensitization. Additionally, we consider the possibility of receptor desensitization. Chronic, low-level leakage of catecholamines from the tumor may have led to the downregulation or desensitization of systemic adrenergic receptors, allowing the patient to adapt to high hormone levels without manifesting classic paroxysmal symptoms.

However, this sequestration presents a latent lethal risk. The stored catecholamines can be explosively released during tumor manipulation. Consequently, this case underscores that biochemistry, not blood pressure, must dictate management. Preoperative alpha-blockade is mandatory for all biochemically active patients, regardless of symptoms. In our case, adequate blockade with doxazosin and an early apical vein ligation technique were instrumental in preventing perioperative instability.

Finally, the loss of SDHB expression carries significant prognostic implications (16, 17). Unlike sporadic cases, SDHB-deficient pheochromocytomas are associated with a higher risk of malignancy and metastasis (18, 19). Therefore, this patient requires a rigorous, lifelong surveillance protocol.

## Conclusion

We demonstrate that SDHB deficiency drives “biochemical-clinical dissociation” through a combination of pseudohypoxia-induced cystic sequestration, potential dopaminergic antagonism, and receptor adaptation. Consequently, biochemistry, not blood pressure, must dictate management: preoperative alpha-blockade is mandatory to prevent lethal crises regardless of symptoms. Furthermore, SDHB loss reclassifies this tumor as a high-risk malignancy, necessitating rigorous lifelong surveillance due to its inherent metastatic potential.

## Data Availability

The original contributions presented in the study are included in the article/supplementary material. Further inquiries can be directed to the corresponding author.
